# Enhancement of bioactive metabolites from solid-state fermentation of Cordyceps fungus using various substrates on ameliorating oxidative stress to liver health

**DOI:** 10.1093/jimb/kuaf012

**Published:** 2025-06-14

**Authors:** Tin Ei Cho, Guoying Zhang, Jianya Ling

**Affiliations:** State Key Laboratory of Microbial Technology, Shandong University, Qingdao 266237, China; School of Pharmacy, Shandong University of Traditional Chinese Medicine, Jinan 250355, China; State Key Laboratory of Microbial Technology, Shandong University, Qingdao 266237, China

**Keywords:** Solid-state fermentation, Cordyceps fungus, Bioactive metabolites, Antioxidant activity, Liver health

## Abstract

*Cordyceps*, a genus of Ascomycetes, represents a group of fungi that has attracted considerable attention from both the scientific community and practitioners of traditional medicine. Extensive research has established that *Cordyceps* exhibits various health-promoting properties, including antioxidant activity and enhanced liver function. Solid-state fermentation (SSF) is recognized as an effective method for cultivating microorganisms on solid substrates. Various optimization strategies for the medium have been employed to improve the production of high-quality bioactive substances. Most research has focused on combining Cordyceps fungi with diverse substrates, including grains, beans, herbal plants, fruits, etc. We explored the potential of these combinations in SSF, highlighting promising methods to increase mycochemical and metabolite yields from Cordyceps fungi, which hold broad application prospects, and the effects of antioxidants and related liver function. This review offers critical insights into effectively incorporating fungi and diverse materials within fermentation processes relevant to the nutritional, pharmacological, and biotechnological sectors.

**One-Sentence Summary:** This review explores the potential of solid-state fermentation (SSF) to enhance the production of bioactive compounds from Cordyceps fungi using various substrates, highlighting its antioxidant and liver health benefits, and providing insights for applications in nutrition, pharmacology, and related industries.

## Graphical Abstract

**Graphical Abstract:**
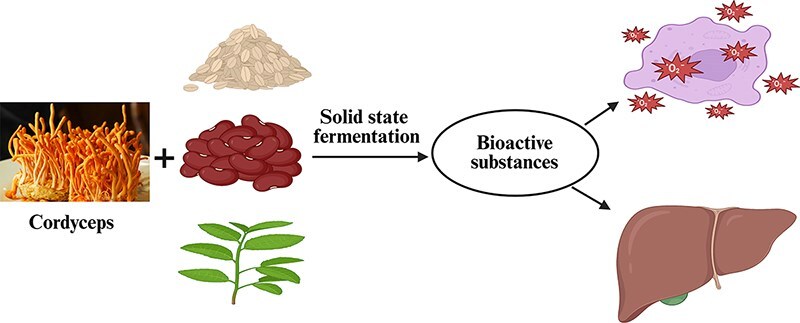

## Introduction

The genus *Cordyceps* encompasses a unique group of medicinal fungi known for their diverse pharmacological properties. Over 750 species of *Cordyceps* have been identified, primarily inhabiting temperate regions at elevations above 3 800 m. Each Cordyceps species relies on a specialized method of survival by invading specific host insects (Olatunji et al., 2018; Sharma et al., 2024). The majority of hosts (more than 95%) in Lepidoptera (moths, butterflies) and Coleoptera (beetles) are larvae; the rest are adults or pupae, making host identification difficult. For example, one of the most popular fungi, *Ophiocordyceps sinensis* (syn. *Cordyceps sinensis*), grows on hepialid larvae in the alpine grasslands of the Himalayas and the Tibetan Plateau (Shrestha et al., 2015). *Cordyceps sinensis* and *Cordyceps militaris* are the most recognized. Owing to their broad spectrum of therapeutic properties, they have been employed in traditional Chinese medicine (TCM) for centuries (Su et al., 2020). *Cordyceps sinensis* demonstrates therapeutic effects on hepatic and renal function and anticancer properties mediated through immune modulation (Chen et al., 2013). Polysaccharides extracted from *C. sinensis* exhibit potent antioxidant activity, offering protective benefits for liver and heart tissues and contributing to overall health (Gan et al., 2012; Liu et al., 2014). *Cordyceps sinensis* has been employed to commercially produce a lot of pharmacological products, such as ‘Golden Sun Cordyceps,’ ‘Ningxinbao,’ and ‘Wanji Cordyceps’ (Dong et al., 2015). Owing to the scarcity of naturally occurring *C. sinensis*, fermented *C. sinensis* is commonly employed as an alternative. This substitute is produced through the purification and fermentation of fungal strains derived from *C. sinensis*. This alternative is recognized in the realm of complementary medical treatment and health products. Fermented *C. sinensis* products adhere to the standards set by the Pharmacopoeia of the People's Republic of China (2015 Edition) (National Pharmacopoeia Committee, 2015). Various studies have highlighted its antioxidant properties (Chen et al., 2013; Yamaguchi et al., 2000a). Extracts from cultured *C. sinensis* demonstrate significant antioxidant and antilipid peroxidation activities and help inhibit the accumulation of cholesteryl esters in macrophages by suppressing low-density lipoprotein (LDL) oxidation (Yamaguchi et al., 2000b; Zhang et al., 2011). Additionally, research indicates that it provides renoprotective effects in diabetic mice, leading to improvements in hyperglycemia and dyslipidemia (Kan et al., 2012; Zhang et al., 2023b). Furthermore, it can mitigate oxidative stress reactions, exhibiting a high degree of selectivity for the heart and liver in its antioxidative effects (Wu et al., 2015). *Cordyceps sinensis* is relatively rare and costly. As a result, *C. militaris* has emerged as a popular alternative due to its comparable pharmacological properties, composition, and lower price (Choi et al., 2014).

*Cordyceps militaris* is increasingly regarded as a viable alternative to Cordyceps fungi in TCM and dietary supplements, owing to their comparable chemical compositions and therapeutic effects (Dong et al., 2012a; Gao et al., 2008; Yue et al., 2008). *Cordyceps militaris* mycelia capsule and powder are authorized commercial pharmaceuticals intended to cure cough and asthma phlegm, nourish the lungs, and energize the kidneys (Dong et al., 2015). *Cordyceps sinensis* and *C. militaris* are commonly used and extensively studied species. *Cordyceps kyushuensis*, closely related to *C. militaris*, also produces both cordycepin and pentostatin (Zhao et al., 2019). Furthermore, *C. kyushuensis* contains numerous pharmacologically active compounds that closely resemble those of *C. sinensis*, making it a viable substitute for this rare fungus (Fan et al., 2006; Li et al., 2002; Wang et al., 2017). The polysaccharides from *C. kyushuensis* possessed high antioxidant effects in *in vitro* system (Zhang et al., 2015) and aqueous extracts of *C. kyushuensis* could contribute to cancer treatment (Zhao et al., 2018). *Cordyceps guangdongensis*, a newly identified species of Cordyceps, was described as emerging from a nature reserve in southern China (Chen et al., 2017). *Cordyceps guangdongensis* contains nutrients and bioactive compounds, including polysaccharides, adenosine, and cordycepic acid. Its nutritional profile is comparable to that of *C. sinensis*. The fruiting bodies of *C. guangdongensis* are recognized for their therapeutic benefits, which include antioxidant activity, antifatigue effects, and anti-inflammatory properties, along with potential advantages for chronic renal failure (Yan et al., 2013, 2014, 2012; Zhang et al., 2018). Additionally, the *C. guangdongensis* fruiting bodies extract has demonstrated efficacy in reducing obesity and associated metabolic disorders (Gangzheng et al., 2023). Furthermore, lipid-lowering compounds extracted from *C. guangdongensis* offer a potential therapeutic formulation for addressing obesity and NAFLD through the modulation of gut microbiota, short-chain fatty acids (SCFAs), and genes associated with fat and lipid metabolism (Wang et al., 2022b). One of the most well-known and ancient TCMs is *Cordyceps cicadae* (Li et al., 2019a). It has shown comparable biological characteristics and bioactive substances to *C. militaris* and *C. sinensis*, indicating that it may be a substitute source of Cordyceps fungi (Nxumalo et al., 2020). This fungus has been utilized as food, a tonic, and in folk medicine to address a variety of ailments, including fever, eye diseases, dizziness, malaria, diabetes, and palpitations. According to reports, crude extracts from *C. cicadae* consist of anticancer, hepatoprotective, and neuroprotective activities (Ke et al., 2018; Li et al., 2019b; Sun et al., 2017; Wang et al., 2018; Xie et al., 2019; Zha et al., 2019). Furthermore, one of the *Cordyceps* species serves as an effective biocontrol agent for controlling different insect populations. It is a *Cordyceps fumosorosea* that is characterized by its broad geographic distribution, high ecological adaptability, ease of cultivation, rapid growth rate, efficient spore production, and extensive application in biological control (Ali et al., 2010). Combining *C. fumosorosea* and *Periplaneta americana* is the best way to find a wide range of bioactive substances (Khan et al., 2024). Over 200 bioactive metabolites have been isolated from various *Cordyceps* species; however, only 35 of approximately 750 recognized *Cordyceps* species have been recorded for their bioactive properties and traditional medicinal uses (Krishna et al., 2024). Researchers are increasingly focusing on edible fungi due to their nutritional and therapeutic potential. Extensive research has demonstrated that the *Cordyceps* genus encompasses a diverse array of biologically active compounds, such as cordycepin, adenosine, polysaccharides, cyclic peptides, cordycepic acids, phenolic compounds, steroids, ergosterol, proteins, fats, carbohydrates, proteoglucans, terpenoids, amphinol, lectins, among others (Ashraf et al., 2020; Zhang et al., 2021). Qu et al. provided a systematic summary of the metabolites derived from the *Cordyceps* genus, summarizing their chemical structures, bioactivities, and potential applications (Qu et al., 2022). These metabolites demonstrate diverse biological activities and health-promoting effects, displaying therapeutic properties including anticancer (Jo et al., 2020), antihyperglycemic (Liu et al., 2023), antifatigue (Hirsch et al., 2017), hepatoprotective (Nguyen et al., 2017), anti-inflammatory (Jiao et al., 2022), antifibrotic (Chen et al., 2012), antioxidant (Wu et al., 2010; Zou et al., 2015), and immunomodulatory activities (Lee et al., 2020). However, natural *Cordyceps* is rare due to its narrow habitat range, host specificity, slow reproduction, overharvesting, and climate vulnerability (Shrestha et al., 2014). As a result, researchers faced significant challenges when attempting to explore these activities in greater depth. A fundamental artificial method for cultivating *Cordyceps* fungi involves using sterile rice media to stimulate stroma formation and facilitate the development of fruiting bodies. The *in vitro* cultivation of *C. militaris* initially relied on insects to grow stromata, which was later advanced through laboratory experiments utilizing various organic substrates. The growth of *Cordyceps* mycelium is influenced by factors such as growth media, temperature, and other environmental conditions. In response to these challenges, researchers have increasingly turned to innovative technologies, such as solid-state fermentation (SSF), to facilitate the large-scale production of bioactive metabolites.

### Solid-State Fermentation

**Fig. 1.**
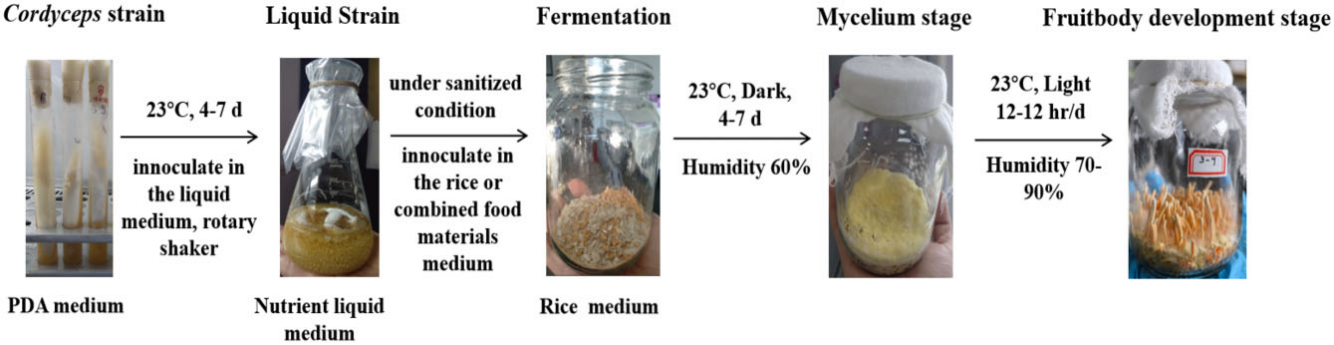
Illustration example of the SSF of *Cordyceps* fungus.

Numerous studies have focused on artificially cultured species. Many of these investigations have produced encouraging results, demonstrating that artificially cultivated Cordyceps fungus exhibits the same potent bioactivities as its wild counterparts (Chen et al., 2009; Dong et al., 2008). Furthermore, various factors significantly impact the growth and quality of Cordyceps fungus in the substrate medium (Turk et al., 2022). Fermentation of Cordyceps fungus, particularly *C. militaris*, can be achieved through two primary methods: submerged fermentation (SmF) and SSF. SmF is a promising alternative to having reasonable control of product quality by maintaining uniform distribution of the substrate, reducing contamination, and diminishing the time of growth (García-Cruz et al., 2019; Tang et al., 2007). SmF is used to produce valuable bio-active metabolites (Petre et al., 2015). However, SmF has its drawbacks, which need to be overcome for optimum production of mycelium from the fungal species (Dudekula et al., 2020). SSF is utilized to create a diverse range of traditional and modern foods. This method is particularly effective for producing various fermented foods, enzymes, and bioactive compounds. Although SSF has historically been used to produce metabolites like enzymes, antibiotics, organic acids, biosurfactants, and aroma compounds, its many uses for achieving metabolite production or remediation goals have drawn more attention (Wang et al., 2010). SSF is recognized as an effective method for cultivating microorganisms on solid substrates. In this process, fungi break down fermentable organic solid materials in the presence of oxygen without adding free water, producing various valuable byproducts. This biotreatment is widely applied in the food sector to enhance the nutritional substrates' profile and enrich them with bioactive compounds, including phenolic compounds with potent antioxidant properties (Feng et al., 2014). Filamentous fungi are particularly well-suited for SSF, as they demonstrate a remarkable tolerance to low water activity, making them the predominant choice in experimental studies (Dulf et al., 2020). A review of the literature indicates that different substrates have been explored for use in SSF, most of which are applicable in tropical countries where local agricultural byproducts can be harnessed. Nevertheless, global cereals, among the most widely cultivated crops globally, are the most commonly used and promising substrates. Their biochemical composition, rich in readily accessible nutrients, promotes the growth and proliferation of microorganisms. The food industry produces a diversity of fermented foods and food ingredients by using SSF. There are enzyme production, fermented foods, bioactive compounds, flavor, and aroma compounds. SSF is employed to produce bioactive compounds like antioxidants, vitamins, and organic acids, which enhance the nutritional value of foods. SSF is a three-stage heterogeneous process that turns a beginning substrate into valuable products by combining solid, liquid, and gaseous phases (Lizardi-Jiménez et al., 2017; Mattedi et al., 2023). SSF offers several advantages, including the production of high volumes, enhanced stability of the final products, greater tolerance to high substrate concentrations, and the use of a natural mix of diverse materials that provide a complete growth medium. Additionally, SSF does not require precise control of the process, allows for easier oxygen supply, and has lower water requirements (Sun et al., 2022). The SSF process consists of preparing a solid medium substrate, inoculating it with the chosen strain, incubating it under controlled conditions, and ultimately harvesting the fungal biomass, as illustrated in Fig. 1. Numerous culture medium optimization strategies have been employed to increase the production of bioactive substances, such as exposure to solid-state medium under UVB and LED radiation, as well as adjustments in pH, temperature, culture duration, and substrate composition (Huang et al., 2017). Some studies have demonstrated that using LED light sources significantly benefits the SSF of Cordyceps fungi, leading to increased productivity of bioactive compounds and improved antioxidant activities (Chiang et al., 2017; Lin et al., 2024). The fermentation environment, including factors like pH, temperature, moisture, and light, serves as a major part in the production of metabolites (Adnan et al., 2017). Most current research has focused on improving growth conditions and media composition to produce a high yield of bioactive compounds and pharmacological effects using a solid culture of Cordyceps fungus. This review provides important information about effectively including fungi and other materials in fermentation processes relevant to the biotechnological, pharmaceutical, and nutritional industries.

### Antioxidant Activity and Liver Health

**Fig. 2.**
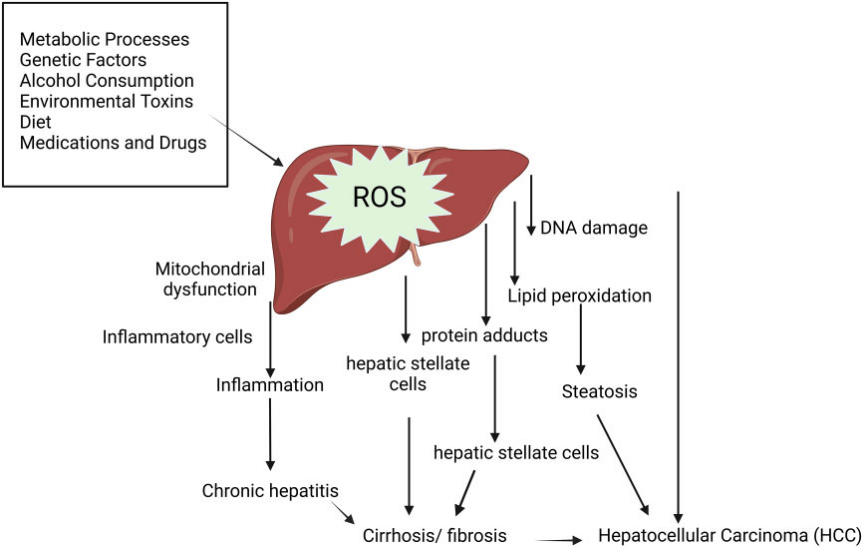
The impact of oxidative stress on hepatic function.

The balancing of oxidative stress is fundamental to liver health. The liver is vital for metabolizing xenobiotics, and generating reactive oxygen species (ROS) is a significant part of this process. An imbalance in ROS production can interfere with liver function and overall metabolic processes. This imbalance happens when the production of ROS surpasses the body's ability to neutralize these harmful byproducts with antioxidants. Excessive ROS can harm essential cellular components such as DNA, proteins, and lipids, ultimately leading to cell death, as shown in Fig. 2. Additionally, oxidative stress can trigger inflammatory pathways, causing liver inflammation and hepatitis. Chronic oxidative stress is associated with the progression of various liver diseases, including alcoholic liver disease, nonalcoholic fatty liver disease (NAFLD), nonalcoholic steatohepatitis, liver fibrosis, cirrhosis, and hepatocellular carcinoma (Apostolova et al., 2011; Cichoż-Lach et al., 2014; Conde de la Rosa et al., 2022). Although oxidative stress represents only one of several factors implicated in liver injury, it is widely acknowledged for its remarkable role in the pathogenesis of liver diseases, especially hepatotoxicity. Hepatocytes, which comprise the majority of liver tissue, play a main role in the metabolism of exogenous chemicals, thereby rendering the liver highly susceptible to the detrimental effects of toxic substances (Hanlon et al., 2009; Kim et al., 2007). Antioxidants are vital in supporting optimal liver function by reducing oxidative stress and inflammation while enhancing the liver's ability to regenerate and repair damaged tissues (Li et al., 2017, Li et al., 2015). These compounds have been widely studied across multiple fields, such as food science, biology, medicine, and nutrition. Many phytochemicals derived from plants, which are categorized as antioxidants, function as reducing agents, scavengers of free radicals, and chelators of metal ions. They play a critical role in mitigating oxidative stress in the human body by preserving the equilibrium between oxidants and antioxidants, thereby reducing the likelihood of diseases associated with oxidative damage and supporting overall health (Chandrasekara et al., 2011; Kim et al., 2012; Wang et al., 2014). Fungi, especially medicinal mushrooms like Cordyceps, are a plentiful source of natural antioxidants. These compounds are crucial in neutralizing ROS and mitigating oxidative stress, which can lead to cellular damage. This protective effect is largely due to their rich content of polyphenols and other antioxidant compounds (Ali et al., 2020; He et al., 2024; Zhang et al., 2023a). Free radicals are unstable molecules that seek stability by extracting electrons from other molecules, leading to a damaging chain reaction (Rahal et al., 2014). Antioxidants effectively neutralize free radicals by donating electrons, thereby halting this destructive process. By counteracting free radicals, antioxidants help maintain balance and prevent oxidative stress. Specifically, Cordyceps fungi extracts have been demonstrated to reduce oxidative stress linked to chronic diseases and ageing, mitigate oxidative damage to the liver, and improve liver function (Deshmukh et al., 2023; Wang et al., 2012).

### Bioactive Substances From Cordyceps Fungus

**Fig. 3.**
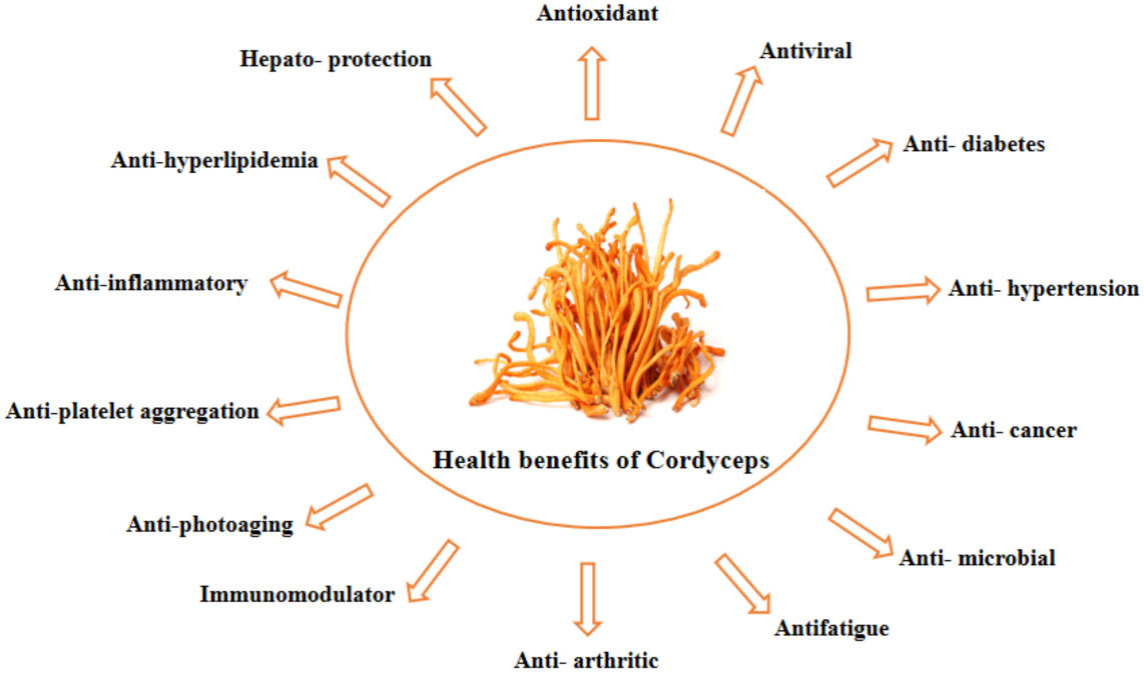
Pharmacological health benefits of Cordyceps fungus in general.

The scientific community is making significant strides in the development of naturally derived products, such as nutraceuticals, aimed at enhancing human health without adverse side effects. The *Cordyceps* genus is known to possess a range of bioactive compounds that offer numerous health benefits (Fig. 3). Research indicates that the *Cordyceps* genus contains various bioactive substances, including cordycepin, adenosine, polysaccharides, phenolic compounds, cordycepic acid, ergosterol, etc. (Tuli et al., 2014; Yang et al., 2014; Yue et al., 2013).

**Fig. 4.**
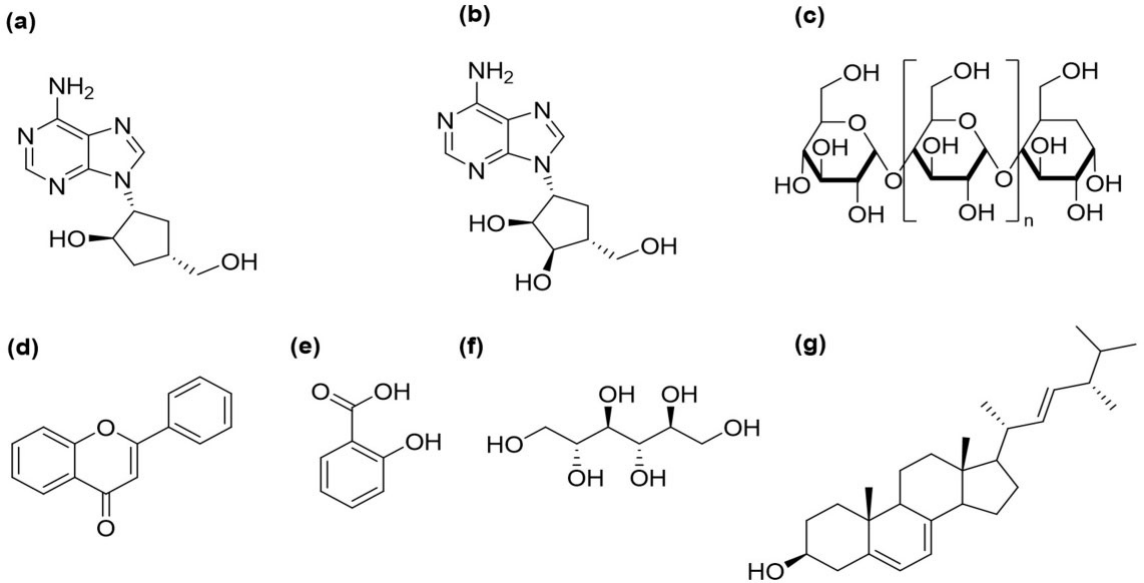
Bioactive compounds from Cordyceps fungus. (a) Cordycepin, (b) Adenosine, (c) Polysaccharide, (d) Flavonoid, (e) Phenolic acid, (f) D-mannitol, (g) Ergosterol.

According to various scientific studies, cordycepin, a bioactive substance derived from Cordyceps fungi, exhibits significant potential for applications across multiple fields and a wide range of biological functions (An et al., 2018; Kondrashov et al., 2012; Kunhorm et al., 2019). Cordycepin, a 3′-deoxyadenosine first isolated from *C. militaris* by Cunningham and colleagues in 1950, can be distinguished from adenosine by lacking a 3′ hydroxyl group (Fig. 4a) (Peng et al., 2024). Naturally occurring in *C. militaris, C. cicadae*, and *C. kyushuensis*, cordycepin has demonstrated therapeutic potential through various activities and research findings (Yoshikawa et al., 2007; Kaczka et al., 1964; Liu et al., 2018; Wang et al., 2022a). Its medicinal properties include antifungal, antimicrobial, anticancer, antidiabetic, antihyperlipidemic, anti-inflammatory, antiplatelet aggregation, antiphotoaging, and immunomodulatory effects (Das et al., 2021; Jiang et al., 2019; Peng et al., 2024). Moreover, *C. militaris* enriched with cordycepin has demonstrated potential in mitigating alcohol-induced hepatotoxicity by boosting the activity of alcohol-metabolizing enzymes and lowering the concentrations of clinically relevant liver enzymes (Cha et al., 2013). Adenosine is a nucleoside comprised of adenine and d-ribose, as depicted in Fig. 4b. It is believed to be a significant active component in the Cordyceps fungus, contributing to reduced neuronal excitability and exhibiting cardioprotective effects (Dong et al., 2012b; Nguyen et al., 2024).

Cordyceps polysaccharides have attracted interest because of their diverse pharmacological characteristics, including their immunomodulatory, anti-inflammatory, anticancer, and antioxidant activities. These polysaccharides are recognized as the primary bioactive constituents in *Cordyceps* in Fig. 4c. They can stimulate the production of immune-active chemicals like cytokines and chemokines while improving the activity of dendritic cells, lymphocytes, and macrophages (Yue et al., 2013; Chen et al., 2024). Additionally, polysaccharide extracts are compatible with existing cosmetic ingredients, making *C. militaris* polysaccharides a preferred choice for skincare formulations (Kanlayavattanakul et al., 2023). Furthermore, polysaccharides derived from *C. militaris* can inhibit adipocyte differentiation and help alleviate hyperlipidemia (Yu et al., 2021). The most significant bioactive ingredients of *C. sinensis* are polysaccharides, which have antiaging, antioxidant, and liver-protective properties. When rats are given CCl4, the exopolysaccharides from the cultivated *C. sinensis* have a hepatoprotective effect against acute hepatotoxicity (Nguyen et al., 2021). The positive health benefits of polyphenols, a significant metabolite found in plants, receive more and more attention. Among their many biological actions are potent antioxidants and the ability to scavenge free radicals (Yu et al., 2018). Flavonoids and phenolic acids are two types of important phenolic compounds present in mushrooms (Fig. 4d, e). Microorganism-derived enzymes are essential for the mobilization of these phenolic compounds. In the bioprocessing of Cordyceps fungus, which efficiently facilitates the production of phenolic acids, the concentration of phenolic compounds rises with the length of SSF (Jedrejko et al., 2021; Wang et al., 2023). Phenolic content and antioxidant activity have been linked in several studies (Dong et al., 2014; Eiamthaworn et al., 2022; Huang et al., 2017). The primary biological activity of phenolic compounds lies in their antioxidant potential, attributed to their ability to transfer electrons or hydrogen atoms due to the specific chemical structure they possess (Victor et al., 2024). Cordycepic acid, derived from the Cordyceps fungus, is often referred to as D-mannitol (Fig. 4f). It is an isomer of quinic acid with a polyol structure and has various potential medicinal applications. The concentration of cordycepic acid varies significantly among different *Cordyceps* species. It stores carbohydrates and helps move other substances, which helps with osmoregulation and metabolic pathway control in *C. militaris* (Cohen et al., 2014; Das et al., 2021; Lin et al., 2016; Shawkat et al., 2012). This substance has antifree radical capabilities, acts as a diuretic, affects plasma osmotic pressure, and is particularly helpful in treating liver fibrosis (Guo et al., 2007; Nomani et al., 2014). Ergosterol is another significant bioactive substance present in the Cordyceps fungus (Fig. 4g). Like cholesterol in animal cells, ergosterol is a sterol that functions as a precursor to vitamin D2 and is necessary for the integrity of the fungal cell membrane (Kitchawalit et al., 2014; Yoneyama et al., 2022).

### Microbial Fermentation for Bioactive Substances

Most studies focus on the microbial fermentation of the *Cordyceps* genus to enhance the bioactive substances, particularly the cordycepin content. In this context, optimizing both medium components and additives is crucial for improving the production of bioactive metabolites. Single-grain substrate media, such as rice, oats, cereals, and wheat, have been utilized for metabolite production (Adnan et al., 2017; Panjikkaran et al., 2013; Yang et al., 2016). Additionally, researchers have sought to optimize the solid-state culture conditions of the Cordyceps fungus to maximize the yield of bioactive metabolites (Lim et al., 2012). Furthermore, the combination of substrate media with different materials such as mung beans, soybeans, chickpeas, black beans, herbal plants, and even fruit has demonstrated enhanced production of metabolites and therapeutic activities, especially antioxidant activities (Table 1). Researchers use many methods to determine the antioxidant activity of the Cordyceps fungus. These include the 2,2-diphenyl-1-picrylhydrazyl(DPPH) Radical Scavenging Assay, Ferric Reducing Antioxidant Power, Hydroxyl Radical Scavenging Assay, Superoxide Anion Radical Scavenging Assay, and Total Phenolic and Flavonoid Content (He et al., 2024; Zhang et al., 2023a; Deshmukh et al., 2023). In the *in vivo* system, it typically evaluates the effects on oxidative stress markers in animal models by measuring the activity of enzymes like superoxide dismutase, glutathione peroxidase, and catalase in blood or tissue samples, lipid peroxidation assay, analyze ROS or reactive nitrogen species levels in biological samples, histopathological analysis, and DNA damage analysis (Yu et al., 2018; Zheng et al., 2014).

**Table 1.** Solid-State Fermentation of Cordyceps Fungi and Different Materials, Metabolites, and Therapeutic Activities

Cordyceps species	Combination materials	Bioactive substances	Biological activities	References
*C. militaris*	Chickpeas	Phenolic, saponin	Antioxidant effects, protective DNA damage	Xiao et al. (2014)
*C. militaris*	Mung bean	Phenolic	Antioxidant effects, protective DNA damage	Xiao et al. (2015)
*C. militaris*	Soybean, chickpea, black bean, mung bean	Cordycepin, phenolic, flavonoid	Antioxidant effects, angiotensin I-converting enzyme (ACE) inhibition	Liu et al. (2022)
*C. militaris*	Rice, wheat, jowar, bajra, sugarcane bagasse	Cordycepin, adenine, adenosine		Borde et al. (2023)
*C. militaris*	Spent brewery grains (SBG)	Cordycepin		Gregori (2014)
*C. militaris*	Vigna radiata hull, houttuynia cordata stem	Polyphenols, polysaccharides	Antioxidant, antimicrobial, immunomodulatory effect	Pi et al. (2024)
*C. kyushuensis*	*Astragalus radix*	Cordycepin, adenosine		Zhang et al. (2021)
*C. militaris*	Chinese jujube fruit	Total phenolic, flavonoid	Antioxidant effects	Wang et al. (2023)
*C. militaris*	Deep ocean water	Cordycepin, adenosine	Antiliver fibrosis	Hung et al. (2017)
*C. Fumosorosea*	*P. americana*	β-1,3-glucan, polysaccharides, cordycepic acid		Khan et al. (2024)
*C. cicadae*	Wheat bran, buckwheat, corn, silkworm chrysalis, yeast	Polysaccharides	Antioxidant, immunomodulatory	Zheng et al. (2022)

### Solid-State Fermentation of Cordyceps and Various Substances

Beans are acknowledged as a source of nutrients with health-promoting qualities and have a multitude of bioactive properties. They contribute to the prevention of numerous chronic conditions, such as heart disease, diabetes, obesity, cancer, and gastrointestinal health issues (Mullins et al., 2021; Singh et al., 2017; Tang et al., 2014). Xiao et al. (2014) were the first to reveal the effects of SSF on chickpeas with *C. militaris* by analyzing the total phenolic and saponin contents, antioxidant activities, and DNA damage protection across different solvent extracts (80% methanol, 80% ethanol, and water). Compared to their unfermented counterparts, the fermented chickpeas had significantly increased levels of total phenolic and saponins and more potent antioxidant and DNA damage protection properties. Additionally, Xiao's research group demonstrated that mung bean SSF using *C. militaris* was remarkably effective in boosting phenolic content, antioxidant activity, and protective properties against DNA damage (Xiao et al., 2015). Interestingly, the maximum phenolic content, DPPH radical scavenging activity, ferric-reducing antioxidant power, reducing power, and DNA damage protection were found in the aqueous extract of *C. militaris*-fermented mung beans. Liu et al. (2022) showed that the legume family, which includes black bean, soybean, chickpea, and mung bean, serves as substrate for SSF with *C. militaris*. The variations in cordycepin levels, as well as the phenolic and flavonoid content, antioxidant activity, and angiotensin I-converting enzyme (ACE) inhibition, are influenced by the different bean samples. Notably, the fermentation period significantly enhances the accumulation of cordycepin, with soybean showing the maximum levels during SSF. Additionally, the total phenolic and flavonoid content showed a marked increase in fermentation time, which corresponded with improved antioxidant activities and ACE inhibition when beans served as the medium for *C. militaris*. Borde et al. (2023) also investigated the effects of various grain combinations on biomass output and *C. militaris* cultivation. According to their findings, the combination of rice, wheat, jowar, and sugarcane bagasse significantly increased the levels of adenine and adenosine during SSF of *C. militaris*. When *C. militaris* was cultivated on rice, wheat, jowar, bajra, and sugarcane bagasse, the most significant amount of cordycepin was found, exceeding the amounts produced by fermentation on rice alone. One of the earliest types of research focused on cultivating *C. militaris* mycelia and fruiting bodies on SSF using spent brewery grains (SBG). As a byproduct, SBG is a readily available and cost-effective solid-state substrate for the production of cordycepin, leading to some of the highest reported concentrations of this compound (Gregori, 2014).

A variety of plant extracts, herbs, and spices are currently under investigation for their antibacterial properties, growth-promoting abilities, and other health benefits (Diaz-Sanchez et al., 2015). Pi et al. (2024) demonstrated the feasibility of using herbal plants: *Vigna radiata* hull and *Houttuynia cordata* stem combined with wheat bran as a substrate for SSF employing *C. militaris*. This fermentation process enhanced the bioactive properties of both *C. militaris* and the herbal matrix, influencing antioxidant and immunomodulatory effects. The resulting fermentation extracts, rich in polyphenols and polysaccharides, exhibited antimicrobial activity against both Gram-positive and Gram-negative bacteria, proving effective as a feed additive in livestock production. Additionally, through biotransformation, fermenting TCM with fungi might increase the concentration and effectiveness of particular active ingredients (Lin et al., 2008). The effects of incorporating *Astragalus radix* into the solid medium used to develop the growth stages of *C. kyushuensis* were investigated by Zhang et al. (2021). The different phases of *C. kyushuensis* were when the fermentation products were obtained. On the 30th day of fermentation, cordycepin and adenosine reached their maximum concentrations in the solid medium supplemented with *Astragalus radix*.

Chinese jujube has a long-standing place in TCM and has been recognized as a dietary supplement food. It was listed among the five most valuable fruits in the ancient text of Huangdi Neijing (475–221 BC), which is a foundational work of Chinese herbal medicine (Agrawal et al., 2023). As a result of its nutritional and functional attributes, such as high levels of polysaccharides as well as phenolic acids and flavonoids, it has demonstrated a lot of pharmacological effects. These include antitumor, antioxidant, anticardiovascular, anti-inflammatory, hepatoprotective, and gastrointestinal effects (Arslan et al., 2021). Wang et al. (2023) conducted a pioneering study on the fermentation of jujube fruit using *C. militaris*. In a solid medium containing 50% jujube, the total phenolic and flavonoid contents rose to optimal levels. Furthermore, zebrafish were protected from oxidative stress by SSF jujube extracts and fermentation extracts demonstrated antioxidant activity in *in vitro* and *in vivo* systems. Furthermore, Hung et al. (2017) investigated the product of SSF of *C. militaris* using deep ocean water (DOW) and its beneficial effects on liver health. The addition of DOW enhanced the functional components of the fungi, leading to improved health benefits. This study revealed that *C. militaris* fermentation with DOW promoted higher levels of cordycepin and adenosine, demonstrating antiliver fibrosis effects through various mechanisms.

The salty and acidic qualities of *Periplaneta americana* are well known for promoting blood circulation, eliminating blood stasis, and improving conditions associated with impaired circulation. It treats bug bites, carbuncles, body phlegm, tonsillitis, sore throats, and infantile malnutrition (Liao et al., 2023). Studies have demonstrated its capacity to minimize liver inflammation, support liver recovery, and decrease the level of liver fibrosis in hepatitis B virus-infected patients (Zhao et al., 2017). *Cordyceps fumosorosea* is a common species in the genus *Cordyceps*. Khan et al. (2024) conducted the first study on cultivating *C. fumosorosea* using *P. americana* as the solid medium. The study focused on enhancing the following bioactive substances: ergosterol, flavonoids, cordycepic acid, polysaccharides, β-1,3-glucan, and nitrogenous compound nucleosides. It was discovered that *C. fumosorosea* mycelia taken after 20 and 25 days were most suited for identifying a range of bioactive substances. *Cordyceps fumosorosea* mycelium and the combination may offer an abundance of bioactive substances with possible health advantages.

Polysaccharides, recognized as the most prominent and physiologically active compounds derived from mushrooms (Wasser, 2002). The cultivation of *C. cicadae* involved transferring the seed culture from a liquid medium to a solid substrate composed of yeast extract, maize flour, buckwheat flour, wheat bran, and silkworm chrysalis powder. Cordyceps polysaccharides demonstrated the ability to alleviate cyclophosphamide-induced toxicity in mice through their immunomodulatory and antioxidant mechanisms (Zheng et al., 2022). These results suggest that cordyceps polysaccharides have potential as a natural immunomodulator, offering therapeutic benefits against oxidative damage and immunosuppression associated with chemotherapy.

## Discussion

This review provides an overview of how oxidative stress impacts liver function, modulates inflammatory pathways, and contributes to different liver diseases. It highlights the potential therapeutic effects of antioxidants from the Cordyceps fungus and enhances bioactive metabolites in the *Cordyceps* fermentation with various substrate materials. Cordyceps is traditionally used in Chinese medicine for its medicinal benefits. Research indicates that Cordyceps may provide many specific benefits for liver health, including antifibrotic properties and hepatoprotective effects. SSF emerges as a pivotal cultivation technique due to its effectiveness in producing bioactive compounds. We explore how SSF optimizes the interplay between *Cordyceps* and diverse substrates, such as grains, beans, herbal plants, and fruits, to enhance the yield of valuable mycochemicals and metabolites. Exploring various combinations and optimization strategies could reveal unique synergies that improve production outcomes and bioactive substance quality. The enhanced production of bioactive substances such as cordycepin, adenine, adenosine, phenolic compounds, flavonoids, polysaccharides, β-1,3-glucan, and cordycepic acid has been observed under SSF of Cordyceps fungi using different substrate combinations. The effects of these bioactive metabolites generated through SSF point to significant potential for increasing the output of valuable compounds with antioxidant properties beneficial for liver health.

### Conclusions and Future Perspectives

Cordyceps fungi are still the focus of much research due to their medical benefits and therapeutic applications. Particularly, *C. sinensis* and *C. militaris* have garnered more attention in recent years. *C. sinensis* is renowned for its ability to treat various ailments with minimal side effects, thanks to its broad range of biologically active substance. However, the overexploitation of this fungus has led to concerns about its sustained longevity in its natural habitat. Compared to *C. sinensis, C. militaris* requires shorter and simpler cultural conditions, making it more accessible for large-scale production. The improvements in biotechnology and sustainable practices are expected to increase their production and effectiveness. The use of diverse substrates has been shown to improve the yield and quality of these bioactive metabolites, known for their therapeutic properties. Further research, experimental and medicinal studies are required to study the combination of food materials and Cordyceps fungus, as well as its effectiveness and safety of the combination. The findings suggest that optimizing the fermentation conditions and substrate combinations can lead to more efficient and cost-effective production processes, making these bioactive substances more accessible for pharmacological and commercial applications.

## Data Availability

Data that support the findings of this research study are available from the corresponding author, upon reasonable.
